# Longitudinal change in occupational engagement configurations in later life: evidence from SHARE France

**DOI:** 10.1016/j.ssmph.2026.101990

**Published:** 2026-10-24

**Authors:** Muriel Sahraoui, Emmanuelle Cambois, Florence Canouï-Poitrine, Marie Laurent, Meoïn Hagege

**Affiliations:** a Université Paris-Est Créteil, Inserm U955, Créteil, F-94010, France; b Institut national d’études démographiques (INED), Mortality, Health and Epidemiology Unit, Campus Condorcet, Aubervilliers, F-93322, France; c Assistance Publique - Hôpitaux de Paris (AP-HP), Hôpital Henri-Mondor, Service Santé Publique, Créteil, F-94010, France

**Keywords:** Life course, Sequence analysis, Everyday activities, Participation, Self-rated health

## Abstract

Later-life participation is often studied in terms of maintenance or decline of individual activities, leaving less clear whether broader configurations of everyday engagement shift uniformly towards more home-centred patterns or undergo more differentiated forms of change. We analysed French data from the Survey of Health, Ageing and Retirement in Europe (SHARE), waves 5-9, to identify configurations of reported activities among adults aged ≥ 50 years and examine their longitudinal transitions. Baseline profiles were derived at wave 5 (N = 4505) using Partitioning Around Medoids with Gower distance and assigned at waves 6–9 using fixed medoids. Transition matrices, sequence indicators, GEE logistic models and multinomial models described trajectories and associations with concurrent health and life-course changes.

Five profiles were identified: Home-centred (73.2%), Family-oriented (9.8%), Community (3.8%), Diversified (7.6%) and Social engagement (5.6%). Home-centred engagement was the most stable profile and the most frequent transition destination. Increased ADL and IADL limitations were associated with higher probabilities of Home-centred engagement. In contrast, retirement was associated with lower Home-centred and higher Community and Diversified engagement, while household-size changes were associated with several profile differences.

Later-life participation therefore did not show a uniform shift towards more home-centred engagement. Instead, different functional and life-course changes were concurrently associated with different multidomain engagement configurations. Home-centred engagement should not be interpreted as evidence of reduced engagement, as the available measures cannot distinguish continuity, adaptation, preference or constraint.

## Highlights

• Five multidomain occupational engagement configurations were identified.
• Profile transitions were asymmetric and frequently towards Home-centred engagement.
• Increased ADL and IADL limitations were associated with Home-centred engagement.
• Retirement was associated with lower Home-centred and higher Community engagement.
• Later-life engagement changes differed across functional and life-course circumstances.

## 1 Introduction

Healthy ageing is increasingly framed as a population health issue concerned with the conditions that enable people to maintain functional ability and to do what they value across later life (WHO, 2021). This perspective shifts attention beyond disease status alone towards the interplay between health, environments, social resources and opportunities for participation. Recent longitudinal evidence has reinforced the importance of social participation and volunteering for health, well-being and functional trajectories in mid and later life, while also showing that participation is embedded in unequal resources and changing life circumstances (Wen et al., 2024; Weziak-Bialowolska et al., 2024). From a population health perspective, engagement in everyday activities is therefore not only an individual behaviour, but a socially patterned dimension of ageing, unequally distributed across health, socioeconomic and relational contexts.

However, much of this literature examines specific participation domains separately, such as volunteering, leisure, family care or social participation. This activity-by-activity approach may overlook the fact that older adults combine multiple activities into broader configurations of everyday life. Recent longitudinal studies have begun to examine changes in specific forms of participation, including volunteering after retirement and the relationship between care provision and social participation in later life (Almeida-Meza et al., 2025; Hämäläinen et al., 2024). Yet less is known about how different domains of engagement are structured together at the population level and how these configurations change over time.

An occupational perspective offers a way to examine how population-level participation indicators combine within broader configurations of everyday activity. In the International Classification of Functioning, Disability and Health (ICF), participation refers to involvement in life situations (WHO, 2001), while occupational approaches additionally emphasise meaning, identity, belonging and continuity (Hammell, 2004; Wilcock, 1999). The indicators available in the Survey of Health, Ageing and Retirement in Europe (SHARE) do not capture these subjective dimensions. The profiles should therefore be understood as configurations of reported everyday activities, representing the observable activity-pattern dimension of occupational engagement rather than its full subjective experience.

Beyond whether individual activities are maintained or lost, an important question is whether change in later-life participation tends towards more home-centred configurations or takes more differentiated forms. Functional limitations may constrain some activities, but they do not themselves determine the form or setting of participation, which also depends on contextual conditions and available opportunities (WHO, 2001). Examining activities as configurations therefore makes it possible to distinguish a general shift towards more home-centred patterns from different forms of change associated with functional, employment and relational circumstances.

A life-course perspective further suggests that these configurations of everyday participation should be considered longitudinally rather than as fixed attributes. Patterns of everyday participation may change with chronological ageing, biographical transitions, linked lives, institutional contexts and cumulative inequalities (Elder, 1994; Settersten, 2003). Retirement, household reorganisation, economic resources and changing family responsibilities may modify both the time available for occupations and the constraints under which participation occurs (Carr & Utz, 2020). Recent SHARE-based evidence has associated the transition to retirement with greater volunteering and community-oriented engagement, particularly among individuals with more favourable health, educational and financial resources (Hämäläinen et al., 2024). Care responsibilities may also interact with social participation in complex ways, reflecting both role extension and role overload depending on care intensity, relationship to the care recipient, gender, resources and care regimes (Almeida-Meza et al., 2025). Functional health is therefore one important dimension to examine alongside changing life-course circumstances. In the present study, it is assessed through limitations in Activities of Daily Living (ADL), which concern basic self-care activities, and Instrumental Activities of Daily Living (IADL), which concern more complex activities required for independent living (Katz et al., 1963; Lawton & Brody, 1969).

The analysis was restricted to France to examine these configurations within a common social and institutional context, consistent with a broader research programme examining later-life engagement through complementary institutional, population-based and biographical approaches.

Against this background, the primary aim of this study was to identify population-level profiles of reported everyday participation among adults aged 50 years and older in France and to describe their longitudinal stability and transitions across SHARE waves 5–9. A secondary aim was to estimate associations between profile membership at the following wave and concurrent changes in selected health and life-course indicators, including self-rated health, ADL and IADL limitations, retirement, household size and economic resources.

## 2 Methods

### 2.1 Data and study population

This study used French data from waves 5 to 9 of the Survey of Health, Ageing and Retirement in Europe (SHARE), a longitudinal survey of adults aged 50 years and older (SHARE-ERIC, 2024a, 2024b, 2024c, 2024d, 2024e; Börsch-Supan et al., 2013).

The observation period covered wave 5 (W5; 2013) to wave 9 (W9; 2021-2022). W5 was selected as the common analytical starting point because it included a refreshment sample and provided a set of activity and participation indicators that could be followed across subsequent waves; it should not be interpreted as the beginning of participants’ occupational trajectories.

Participants were required to be observed at W5 and to contribute the information necessary to construct an occupational engagement profile. Longitudinal analyses additionally required at least two consecutive observations to contribute a transition. Respondents entering SHARE after W5 were therefore not included in the longitudinal framework. These criteria may preferentially retain individuals with more stable survey participation and are considered as a potential source of selection bias. Proxy participation was not an exclusion criterion; participants were retained when the data required for the relevant analyses were available.

Data collection during W8 and W9 was affected by the COVID-19 pandemic and changes in interview procedures. To preserve comparability of the information used to construct and follow the profiles, the analysis was restricted to interviews conducted using Computer-Assisted Personal Interviewing (CAPI) (Bergmann & Börsch-Supan, 2021; Bergmann et al., 2024). This restriction may itself have introduced additional selection during the pandemic period if the ability to participate through CAPI differed according to health, living conditions or vulnerability. Profile information was available for N = 4505 participants at W5, W6 and W7, N = 2603 at W8 and N = 1937 at W9; N = 1814 had profiles at all five waves and contributed to complete-sequence analyses. Attrition and the potential influence of this selection were examined in sensitivity analyses using inverse probability weighting.

Individuals with more than 20% missing data on variables used to construct the occupational engagement profiles were excluded from profile construction (Bennett, 2001). Refusals, “don't know” responses and other item non-responses were treated as ordinary missing data, whereas missing values resulting from conditional questionnaire routing were treated as structural missingness and were not interpreted as absence of the corresponding activity. Remaining missing values were not imputed. Consequently, analytical sample sizes could vary across variables and models according to data availability.

### 2.2 Occupational engagement profiles

Profiles were constructed from SHARE indicators of social, domestic, family, productive, community, cultural, leisure and physical activities, organised using the ICF as a conceptual framework (Table 1). Indicators captured activity diversity and participation frequency through ordinal, binary and categorical variables. Simple frequency items were coded from never (0) to daily (4), while three composite ordinal indicators were derived in the analytic dataset as described in Table 1.

**Table 1:** Activities and characteristics of occupational engagement profiles (France, SHARE wave 5).

Variables		Total N = 4505	P1 (73.2%)	P2 (9.8%)	P3 (3.8%)	P4 (7.6%)	P5 (5.6%)	ICF Domain
**Panel A. Constituent activities used to derive occupational engagement profile**								
Providing help to a non-household member, median (0-4)		0	0	0	4	3	1	d6
Providing help to a household member, median (0-4)		0	0	2	0	0	0	d6
Contact with parents, median (0-4)		3	2	3	4	3	1	d7
Grandchild care, median (0-4)		1	0	1	1	2	1	d7
Employment status, n (%)								d8
	Employed	1084 (24.6)	617 (19.2)	163 (37.7)	64 (38.3)	158 (46.2)	82 (33.2)	
	Retired	2880 (65.4)	2288 (71.1)	210 (48.6)	84 (50.3)	159 (46.5)	139 (56.3)	
	Other	443 (10.1)	314 (9.8)	59 (13.7)	19 (11.4)	25 (7.3)	26 (10.5)	
Volunteering/unpaid voluntary work, median (0-4)		2	1	2	3	3	3	d9
Education/training courses, median (0-4)		0	0	1	1	2	1	d9
Sports club or social club participation, median (0-4)		3	1	3	3	3	3	d9
Political activities or associations, median (0-4)		0	0	1	1	2	1	d9
Reading, median (0-4)		4	4	4	4	4	4	d9
Word or number games, median (0-4)		4	4	3	4	4	4	d9
Card games or chess, median (0-4)		2	2	2	2	2	2	d9
Moderate physical activity at least once a week (binary), % yes		81.0	78.0	93.2	87.1	100.0	89.0	d9
**Panel B. Characteristics of occupational profiles**								**Between-profile test**
Age, years, mean (SD)		67.6 (10.7)	69.6 (10.8)	61.9 (7.6)	61.1 (7.3)	61.4 (7.7)	64.2 (10.2)	F = 122.04, p < .001
Number of ADL limitations, median [IQR]		0 [0-0]	0 [0-0]	0 [0-0]	0 [0-0]	0 [0-0]	0 [0-0]	H(4) = 98.52, p < .001
Any ADL limitation, %		13.1	16.1	5.2	3.5	3.5	7.2	**χ^2^**(4) = 98.92, p < .001
Number of IADL limitations, median [IQR]		0 [0-0]	0 [0-0]	0 [0-0]	0 [0-0]	0 [0-0]	0 [0-0]	H(4) = 132.61, p < .001
Any IADL limitation, %		17.8	21.5	6.4	8.8	4.1	14.7	**χ^2^**(4) = 125.54, p < .001
Number of chronic conditions, mean (SD)		1.7 (1.5)	1.8 (1.5)	1.3 (1.2)	1.2 (1.2)	1.2 (1.2)	1.6 (1.5)	F = 27.73, p < .001
Good to excellent self-rated health, %		63.1	58.6	75.2	77.8	81.7	65.9	χ^2^(4) = 124.78, p < .001
Low education level (ISCED 0–2), %		43.2	49.0	32.0	23.0	29.0	37.0	χ^2^(12) = 444, p < .001
Women, %		57.4	56.0	65.1	71.1	56.0	53.0	χ^2^(4) = 32.6, p < .001
Rural area or village (%)		47.6	48.0	48.6	47.7	47.2	43.4	χ^2^(4) = 2.78, p = .595
Economic difficulty score, mean (SD)		3.1 (1.8)	3.3 (2.9)	2.7 (2.0)	3.1 (2.3)	2.8 (2.4)	3.0 (2.4)	F = 25.4 p < .001

**Note.** Panel A reports the constituent activities used to derive occupational engagement profiles and is descriptive only. Median values are reported for ordinal variables, percentages for binary variables, and n (%) for categorical variables. Three activity indicators are composite ordinal scores derived in the analytic dataset (help to a non-household member, contact with parents, and grandchild care); their scale therefore differs from simple 0–4 frequency items. Panel B reports profile characteristics. Continuous variables are presented as mean (SD), skewed discrete variables as median [IQR], and categorical variables as percentages. Because ADL and IADL showed marked floor effects, the proportion of participants reporting at least one limitation is also presented. Between-profile comparisons were assessed using ANOVA for continuous variables, Kruskal–Wallis tests for ordinal or skewed discrete variables, and chi-square tests for categorical variables. Sample sizes vary across variables because of item non-response. ICF = International Classification of Functioning, Disability and Health; ADL = Activities of Daily Living; IADL = Instrumental Activities of Daily Living.

W5 profiles were identified using Partitioning Around Medoids (PAM) with Gower dissimilarity to accommodate mixed variable types (Kaufman & Rousseeuw, 1990). To prevent domains represented by more questionnaire items from dominating the solution, each ICF domain contributed an equal total weight: the two d6 and two d7 indicators each received a weight of 1/2, the single d8 indicator a weight of 1, and the eight d9 indicators a weight of 1/8.

Solutions from two to ten profiles were compared using average silhouette width (ASW), Calinski–Harabasz (CH), Davies–Bouldin (DB), R^2^ and Pakhira–Bandyopadhyay–Maulik (PBM) indices (Calinski & Harabasz, 1974; Davies & Bouldin, 1979; Pakhira et al., 2004; Rousseeuw, 1987). The two-profile solution had the highest ASW (.512) and PBM (9.858) but explained 18% of the variance; the four-profile solution had the lowest DB (2.586); and the five-profile solution had the highest CH (482.750), with ASW = .412 and R^2^ = .43. Solutions with six or more profiles increased explained variance at the cost of lower cohesion and greater fragmentation. Five profiles were therefore retained as a compromise between statistical quality and empirical differentiation (Supplementary Table S1).

At W6–W9, indicators were coded and weighted identically, and each observation was assigned to the closest W5 medoid. Fixed medoids provided a common longitudinal reference, while assuming that the W5 configurations remained relevant. Assignment diagnostics showed modest changes over time: median distance to the closest medoid increased from .142 at W5 to .160 at W9, and the median ratio between the closest and second-closest medoid distances increased from .61 to .66 (Supplementary Table S2).

### 2.3 Variables

Self-rated health was included because it captures perceived health deterioration beyond diagnosed conditions and is a robust indicator of later-life health (Reinwarth et al., 2024). Self-rated health was assessed using the SHARE general health question and recoded into three categories: good to excellent, fair, and poor to very poor. Deterioration was defined as a movement to a less favourable category between t and t+1. Chronic morbidity was measured as the number of chronic conditions reported using the standardised SHARE list.

Functional limitations were measured using Activities of Daily Living (ADL) and Instrumental Activities of Daily Living (IADL) counts, each representing the number of individual activities for which a limitation was reported. To ensure comparability across waves, the IADL count was restricted to items assessed consistently from W5 to W9. Functional deterioration was defined as an increase in the ADL or IADL count between t and t+1; stable or decreased counts formed the reference category. These indicators therefore capture worsening functional limitation rather than first onset among previously limitation-free participants. Because functional and profile changes were measured over the same wave interval, the analyses estimate concurrent longitudinal associations without establishing their temporal ordering within that interval.

Sociodemographic variables included age, sex and education. Age was treated as a continuous, time-varying variable. The SHARE variable gender was coded as men/women and used as the sex indicator in the analyses. Education was measured using the International Standard Classification of Education (ISCED-97) and grouped into four categories: low (0–2), medium (3–4), short tertiary (5), and long tertiary (6). Education was used as the main socioeconomic covariate because educational attainment is generally established by early adulthood and remains relatively stable thereafter, making it a useful indicator of socioeconomic position across the adult life course (Galobardes et al., 2006). Economic difficulties were examined separately as a time-varying measure of current material resources.

### 2.4 Life events and time-varying resources

Life-course and contextual changes were measured between consecutive waves. Retirement was defined as a transition from employment to retirement. Change in household size was measured by comparing the number of people living in the household at t and t+1 and was classified as stable, increased or decreased. This indicator captures a change in the immediate co-residential context rather than the specific biographical event underlying that change. A decrease may result, for example, from bereavement, separation, a child leaving the household or another residential change, while an increase may also reflect different relational or residential events. These situations were therefore not interpreted as equivalent life-course events. The indicator was used more narrowly to examine whether changes in the number of people sharing the household were associated with changes in activity configurations (Carr & Utz, 2020).

Economic resources were measured using a composite score based on eight SHARE “afford to” items, ranging from 0, no economic difficulty, to 8, severe economic deprivation; changes were defined as variations in this score between waves. A comparable score could be constructed at W5, W6, W8 and W9; economic change was therefore analysed only for W5→W6 and W8→W9.

### 2.5 Statistical analysis

Analyses proceeded in three steps. Each individual could contribute several observations corresponding to successive transitions between waves, creating intra-individual dependence that was accounted for in the longitudinal regression models.

First, W5 profiles were described using medians, means and percentages, with chi-square tests, ANOVA or Kruskal-Wallis tests as appropriate. Because activity indicators were used to construct the profiles, their distribution across profiles was interpreted descriptively rather than as external inferential comparisons.

Second, longitudinal trajectories were described using transition matrices and sequence analysis. Complete sequences for N = 1814 participants with profiles at W5–W9 were analysed in R with TraMineR (Gabadinho et al., 2011; Studer & Ritschard, 2016), using the number of distinct states, number of between-wave changes, full-sequence stability and normalised entropy.

Third, overall profile mobility was defined as changing versus remaining in the same profile between consecutive waves and estimated using a population-averaged logistic GEE model with an exchangeable working correlation and robust standard errors (Liang & Zeger, 1986). The model included profile of origin, age, sex, education, ADL and IADL counts, and survey wave.

Transition orientation was analysed using longitudinal multinomial logistic regression models, with profile membership at t+1 as the dependent variable and profile of origin included in each model. Each time-varying change was first examined in a separate model adjusted for profile of origin. These models were intended to estimate the association between each measured change and profile membership at t+1, rather than its association independently of other changes occurring over the same interval. Estimates from these models were therefore not interpreted as mutually adjusted effects. Robust standard errors were clustered at the individual level. Results from multinomial models were expressed as average marginal effects (AME) in percentage points. P-values are reported for all estimates, with 95% confidence intervals for the health models.

A complementary multinomial model included deterioration in self-rated health and increases in the number of ADL and IADL limitations simultaneously, allowing their associations with profile membership to be examined when the three health dimensions were represented jointly. A supplementary binary logistic model examined entry into Family-oriented engagement among transitions originating from any of the other four profiles. The model included sex, age, ADL and IADL counts, profile of origin and survey wave, with standard errors clustered at the individual level. Results were expressed as average marginal effects.

Inverse probability weighting (IPW) was used in sensitivity analyses for differential follow-up. Retention probabilities for W7→W8 and W8→W9 were estimated from age, sex, education, W5 ADL and IADL limitations, profile of origin and self-rated health at the origin wave. Stabilised weights were used for W7→W8 and accumulated for W8→W9; W5→W6 and W6→W7 received weight 1. No weight truncation was applied (Seaman & White, 2013).

Weighted Gower dissimilarities and PAM clustering were implemented in R 4.3 using the *daisy* and *pam* functions of the cluster package. Sequence analysis was conducted using TraMineR, including *seqdef* and *seqtrate* for sequence definition and transition summaries. Longitudinal multinomial logistic models were estimated in Stata/SE 18.0 using *mlogit*, and average marginal effects were obtained using *margins*. The population-averaged logistic GEE model was estimated with a binomial distribution, logit link, exchangeable working correlation structure and robust standard errors. A two-sided alpha level of 5% was used.

## 3 Results

### 3.1 Sample and occupational engagement profiles at baseline

At wave 5, the analytical sample included 4505 individuals living in France. Mean age was 67.6 years and 57.4% were women. Five occupational engagement profiles were identified at baseline. Because profiles were derived from participation indicators, they are first described according to their activity configurations; sociodemographic, economic and health characteristics are presented as external profile characteristics (Table 1).

Home-centred engagement was the largest profile (73.2%) of the sample. It was mainly centred on individual and home-based activities, including reading, cognitive games and, to a lesser extent, card games or chess. In contrast, community participation, volunteering, educational activities, help to others and childcare were limited. Participants in this profile were older, less educated and showed less favourable health characteristics than participants in most other profiles, with more chronic conditions, poorer self-rated health and more frequent ADL and IADL limitations (Table 1).

Family-oriented engagement (9.8%) was characterised by family-oriented activities, particularly frequent contact with parents and help within the household, combined with some club participation, volunteering and cognitive activities. Participants in this profile were younger than those in Home-centred engagement, predominantly women, and generally had fewer functional limitations (Table 1).

Community engagement (3.8%) combined frequent family contact with extra-household help, volunteering and club participation. Reading and cognitive activities were also frequent. Participants in this profile were relatively young, predominantly women, and had generally favourable health and educational characteristics (Table 1).

Diversified engagement (7.6%) was characterised by the broadest combination of measured activity domains, including club participation, volunteering, educational or political activities, childcare, help outside the household and moderate physical activity. Participants in this profile were, on average, the youngest, had the highest proportion still employed, the most favourable health characteristics and relatively high educational resources (Table 1).

Social engagement (5.6%) was characterised by sustained social, cultural and volunteering activities, but a less diversified pattern and with less-family-oriented and physical activity than Diversified engagement. Participants in this profile had intermediate sociodemographic and health characteristics compared with the other profiles (Table 1).

### 3.2 Longitudinal trajectories and profile mobility

Between W5 and W9, trajectories showed an asymmetrical pattern of stability and change (Fig. 1, Table 2). Home-centred engagement was substantially more stable than the other profiles: 57.3% of participants initially in this profile remained there at the end of follow-up, compared with 7.0%–17.5% for the other four profiles. Transitions were predominantly directed towards Home-centred engagement, whereas transitions out of this profile and direct movements between highly contrasted configurations were less frequent.

**Fig. 1:**
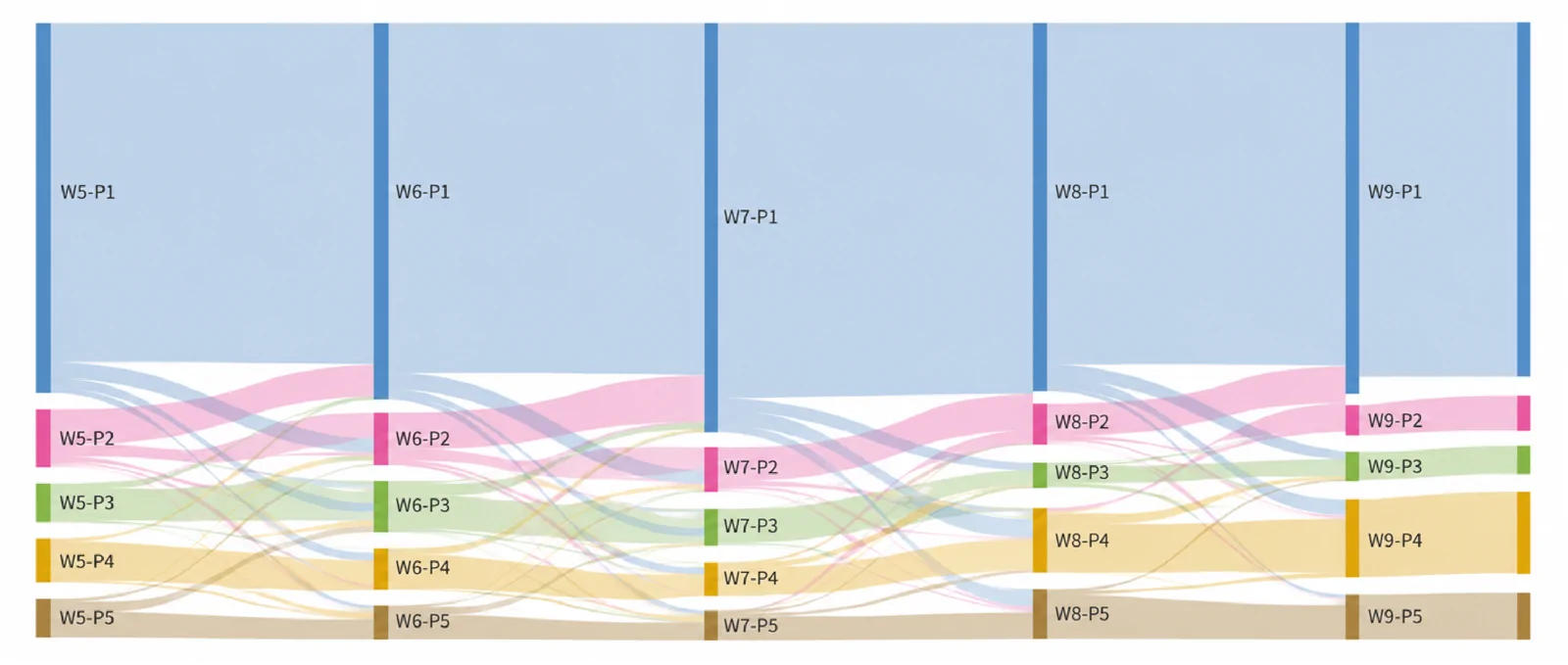
Longitudinal transitions between occupational engagement profiles among individuals aged 50 years and older in France (SHARE, waves 5–9). **Note**: W denotes survey wave and P denotes occupational engagement profile. Each column corresponds to one survey wave (W5 to W9). Rectangles represent the identified occupational engagement profiles, with their width proportional to the share of individuals belonging to each profile at the given wave. Flows between columns indicate individual transitions between profiles from one wave to the next, and flow thickness is proportional to the observed frequency of transitions. For readability, profile codes correspond to P1 = Home-centred engagement, P2 = Family-oriented engagement, P3 = Community engagement, P4 = Diversified engagement, and P5 = Social engagement.

**Table 2:** Observed transitions between occupational engagement profiles across consecutive waves (W5 W9).

Profile at t	P1 Home-centred engagement	P2 Family-oriented engagement	P3 Community engagement	P4 Diversified engagement	P5 Social engagement	Total
**Panel A. Pooled transition matrix across consecutive waves**						
P1 Home-centred engagement	8428 (87.3%)	154 (1.6%)	286 (3.0%)	446 (4.6%)	338 (3.5%)	9652 (100%)
P2 Family-oriented engagement	410 (34.4%)	323 (27.1%)	142 (11.9%)	178 (14.9%)	139 (11.7%)	1192 (100%)
P3 Community engagement	416 (54.5%)	106 (13.9%)	87 (11.4%)	119 (15.6%)	35 (4.6%)	763 (100%)
P4 Diversified engagement	471 (43.8%)	217 (20.2%)	119 (11.1%)	201 (18.7%)	67 (6.2%)	1075 (100%)
P5 Social engagement	440 (59.1%)	112 (15.0%)	46 (6.2%)	87 (11.7%)	60 (8.1%)	745 (100%)
Total	10,165 (75.7%)	912 (6.8%)	680 (5.1%)	1031 (7.7%)	639 (4.8%)	13,427 (100%)
**Panel B. Probability of remaining in the same profile by wave interval**						
W5→W6	84.7%	29.3%	17.5%	19.8%	6.8%	67.4%
W6→W7	95.5%	28.8%	10.9%	8.1%	5.9%	71.8%
W7→W8	80.6%	19.2%	6.8%	31.1%	10.2%	63.9%
W8→W9	83.5%	30.8%	8.5%	24.3%	11.6%	64.1%

**Note.** Values are n (row %). Each row represents the profile observed at the beginning of a transition between two consecutive waves. The total refers to transition observations rather than distinct individuals.

**Note.** Profile-specific percentages correspond to the diagonal of the transition matrix for each wave interval. Overall stability is the proportion of all observations remaining in the same profile between two consecutive waves.

Among the 1814 participants with complete W5–W9 sequences, 33.8% remained in the same profile across all five waves. Full-sequence stability was concentrated in Home-centred engagement (50.8%), whereas it was rare or absent among the other baseline profiles (Supplementary Table S4).

Longitudinal GEE models confirmed this asymmetry (Table 3). Compared with Home-centred engagement, originating from any of the other profiles was associated with a substantially higher probability of profile change (+35.50 to +47.10 percentage points; all p < .001). Mobility decreased slightly with age (−.10 percentage points per year, 95% CI [−.20, −.01], p = .035) and was lower among women (−3.30 percentage points, 95% CI [−5.00, −1.60], p < .001). Higher ADL and IADL counts were also associated with lower mobility (−2.20 and −4.50 percentage points per additional limitation, respectively; p = .003 and p < .001). Short tertiary education was associated with a small increase in mobility (+2.50 percentage points, p = .035), whereas medium and long tertiary education were not statistically significantly associated with profile change. Results were similar in the IPW sensitivity analysis (Supplementary Table S6, Panel B).

**Table 3:** Average marginal effects associated with the probability of changing occupational engagement profile between two consecutive waves (W5–W9).

Variable	AME (percentage points)	95% CI	p
**Profile of origin**			
P1 Home-centred engagement	Reference	—	—
P2 Family-oriented engagement	+40.90	[38.10, 43.70]	<.001
P3 Community engagement	+44.60	[41.30, 47.90]	<.001
P4 Diversified engagement	+35.50	[32.30, 38.70]	<.001
P5 Social engagement	+47.10	[44.30, 49.90]	<.001
**Individual characteristics**			
Age, per additional year	−.10	[−.20, −.01]	.035
Women vs men	−3.30	[−5.00, −1.60]	<.001
**Education**			
Low	Reference	—	—
Medium	+1.90	[−.20, 3.90]	.077
Short tertiary	+2.50	[.20, 4.80]	.035
Long tertiary	+2.20	[−2.40, 6.90]	.350
ADL limitations, per additional limitation	−2.20	[−3.60, −.70]	.003
IADL limitations, per additional limitation	−4.50	[−5.40, −3.60]	<.001

**Note.** AME = average marginal effect, expressed in percentage points. The outcome was profile change versus stability between consecutive waves. Estimates were obtained from a population-averaged logistic GEE model adjusted for profile of origin, age, sex, education, ADL and IADL counts, and survey wave.

### 3.3 Entry into family-oriented engagement

Family-oriented engagement was more frequent among women at W5, who represented 65% of this profile compared with 57.4% of the overall sample. A complementary longitudinal analysis examined entry into Family-oriented engagement among transitions originating from any of the other four profiles.

Among transitions originating from the other four profiles, women had a slightly higher adjusted probability of entering Family-oriented engagement than men (25.17% vs 23.48%; AME = +1.70 percentage points, 95% CI [.13, 3.27], p = .034). Entry was slightly less frequent with increasing age (−.12 percentage points per year, p = .004) and with higher ADL and IADL counts (−1.75 and −3.51 percentage points per additional limitation; p = .031 and p < .001, respectively; Supplementary Table S5).

Thus, entry into Family-oriented engagement was associated with both sex and functional characteristics, although the difference between women and men was modest.

### 3.4 Health changes and transition orientation

Health changes over a wave interval were associated with profile membership at t+1 (Table 4). Deterioration in self-rated health was associated with a higher probability of Home-centred engagement (+7.61 percentage points, 95% CI [4.83, 10.39], p < .001), as were increases in ADL (+17.76 percentage points, 95% CI [14.51, 21.00], p < .001) and IADL limitations (+22.37 percentage points, 95% CI [19.66, 25.07], p < .001). These changes were accompanied by lower probabilities of membership in several of the other profiles.

**Table 4:** Average marginal effects for health and life-course changes on occupational engagement profile membership at t+1.

Change between t and t+1	N	P1 Home-centred	P2 Family-oriented	P3 Community	P4 Diversified	P5 Social
Deterioration in self-rated health	9072	+7.61 [4.83, 10.39] (<.001)	−.36 [−2.68, 1.97] (.764)	−2.14 [−3.47, −.81] (.002)	−3.41 [−5.01, −1.82] (<.001)	−1.70 [−2.99, −.42] (.010)
Increase in ADL limitations	9044	+17.76 [14.51, 21.00] (<.001)	−7.22 [−9.84, −4.61] (<.001)	−3.15 [−4.68, −1.63] (<.001)	−5.31 [−7.09, −3.53] (<.001)	−2.07 [−3.62, −.51] (.009)
Increase in IADL limitations	9050	+22.37 [19.66, 25.07] (<.001)	−10.37 [−12.46, −8.28] (<.001)	−2.69 [−4.07, −1.31] (<.001)	−6.02 [−7.52, −4.53] (<.001)	−3.28 [−4.51, −2.06] (<.001)
Transition from employment to retirement	8844	−12.76 (<.001)	−.18 (.931)	+8.17 (<.001)	+3.97 (.017)	+.80 (.525)
Decrease in household size	13,300	+7.21 (<.001)	−3.00 (.033)	−2.22 (.002)	−2.00 (.034)	+.01 (.987)
Increase in household size	13,300	+38.45 (<.001)	+9.80 (.019)	−15.19 (<.001)	−18.12 (<.001)	−14.93 (<.001)
Increase in economic difficulties	3464	−2.33 (.345)	−1.78 (.673)	+.82 (.754)	+3.44 (.125)	−.15 (.930)

**Note.** Values are average marginal effects in percentage points. For health-change models, 95% confidence intervals are reported in square brackets and p-values in parentheses; for life-course and economic models, p-values are reported in parentheses. Each change was examined in a separate longitudinal multinomial model adjusted for profile of origin, with robust standard errors clustered at the individual level. The economic-difficulty model was additionally adjusted for survey wave and restricted to W5→W6 and W8→W9. Health, life-course and profile changes were measured over the same wave interval; their temporal ordering within that interval cannot be established. IPW sensitivity estimates are reported in Supplementary Table S6, Panel C.

When the three health changes were included simultaneously, deterioration in self-rated health was no longer statistically significantly associated with Home-centred engagement (AME = +.20 percentage points, 95% CI [−3.40, 3.80], p = .930). Increases in ADL (+10.80 percentage points, 95% CI [6.30, 15.30], p < .001) and IADL limitations (+20.80 percentage points, 95% CI [17.10, 24.50], p < .001) remained associated with a higher probability of Home-centred engagement (Table 5).

**Table 5:** Average marginal effects for jointly modelled health changes in a model of occupational engagement profile membership at t+1.

Health change	Profile at t+1	AME (percentage points)	95% CI	p
**Deterioration in self-rated health**	P1 Home-centred engagement	+.20	[−3.40, 3.80]	.930
	P2 Family-oriented engagement	+4.60	[1.10, 8.60]	.009
	P3 Community engagement	−2.20	[−4.10, −.20]	.029
	P4 Diversified engagement	−2.20	[−4.40, −.10]	.045
	P5 Social engagement	−.30	[−2.20, 1.50]	.730
**Increase in ADL limitations**	P1 Home-centred engagement	+10.80	[6.30, 15.30]	<.001
	P2 Family-oriented engagement	−3.70	[−7.60, .20]	.064
	P3 Community engagement	−2.50	[−4.90, −.10]	.039
	P4 Diversified engagement	−3.50	[−6.10, −.90]	.009
	P5 Social engagement	−1.10	[−3.50, 1.30]	.360
**Increase in IADL limitations**	P1 Home-centred engagement	+20.80	[17.10, 24.50]	<.001
	P2 Family-oriented engagement	−10.40	[−13.40, −7.40]	<.001
	P3 Community engagement	−3.60	[−5.40, −1.70]	<.001
	P4 Diversified engagement	−4.00	[−6.20, −1.90]	<.001
	P5 Social engagement	−2.80	[−4.50, −1.00]	.002

**Note.** AME = average marginal effect, expressed in percentage points. The multinomial model simultaneously included deterioration in self-rated health and increases in ADL and IADL limitations and was adjusted for profile of origin, with robust standard errors clustered at the individual level. Health changes and profile changes were measured over the same interval; their temporal ordering within that interval cannot be established. N = 9037 observations from 3330 participants.

### 3.5 Retirement, household size and economic resources

Retirement and changes in household size were also associated with profile membership at t+1 (Table 4). Transition from employment to retirement was associated with a lower probability of Home-centred engagement (−12.76 percentage points, p < .001) and higher probabilities of Community (+8.17 percentage points, p < .001) and Diversified engagement (+3.97 percentage points, p = .017).

Changes in household size showed a broader pattern. A decrease was associated with a higher probability of Home-centred engagement (+7.21 percentage points, p < .001) and lower probabilities of Family-oriented, Community and Diversified engagement. An increase was associated with substantially higher probabilities of Home-centred (+38.45 percentage points, p < .001) and Family-oriented engagement (+9.80 percentage points, p = .019), and lower probabilities of the other three profiles. The underlying biographical events responsible for these household-size changes were not identified.

Increased economic difficulties were not statistically significantly associated with membership in any profile (all p > .05). These life-course and economic changes were examined in separate models and should therefore not be interpreted as mutually adjusted effects.

IPW sensitivity analyses yielded broadly similar patterns of association (Supplementary Table S6, Panel C).

## 4 Discussion

This study identified five configurations of reported everyday activities among French participants in SHARE and showed marked asymmetry in their longitudinal trajectories. Home-centred engagement accounted for nearly three quarters of the W5 sample, was substantially more stable than the other profiles and was the most frequent destination of transitions. Functional deterioration, retirement and changes in household size were associated with different distributions of these configurations. Taken together, these results indicate that longitudinal change in observable participation was patterned, but not uniformly oriented in the same direction.

Theoretically, this pattern supports understanding later-life participation as changing configurations of everyday engagement rather than as a uniform narrowing of participation with advancing age. Functional capacity represents one dimension associated with these configurations but does not account for their overall longitudinal pattern. The contribution of the profile-based approach therefore lies not simply in showing that worsening function is associated with more Home-centred engagement, but in showing that domains of everyday participation combine and change differently across functional and life-course circumstances. This perspective also cautions against treating Home-centred engagement as synonymous with reduced engagement or as an inevitable outcome of ageing. Instead, its longitudinal significance depends on the broader functional, relational and life-course context in which that configuration occurs.

### 4.1 Functional health and the interpretation of home-centred engagement

The health analyses distinguish perceived health from functional limitations. When self-rated health, ADL and IADL changes were considered simultaneously, deterioration in self-rated health was no longer statistically significantly associated with Home-centred engagement, whereas increases in ADL and particularly IADL limitations remained associated with this profile. ADL concern basic self-care, while IADL capture more complex activities required for independent living and are generally affected earlier in functional decline (De Carvalho Yokota & Van Oyen, 2020; Katz et al., 1963; Lawton & Brody, 1969). The stronger association with IADL limitations may therefore reflect the relationship between functional capacity and the practical conditions under which complex everyday activities remain practicable. Because functional and profile changes were measured over the same wave interval, however, this pattern should be interpreted as a concurrent association rather than as evidence that functional deterioration preceded profile change or that specific mechanisms such as transport, fatigue or environmental accessibility were involved.

The intuitive plausibility of this association should not, however, make Home-centred engagement appear to be an inevitable consequence of functional limitation. Functional capacity is one condition of participation but does not alone determine where or how everyday engagement occurs. From an ICF perspective, participation also depends on contextual conditions and available opportunities. A greater probability of Home-centred engagement among participants with increasing functional limitations may therefore coexist with very different environmental, relational and practical circumstances. Treating this pattern as self-evident in later life risks overlooking the conditions under which participation remains possible beyond the home.

This distinction is important because Home-centred engagement occupied a distinctive longitudinal position: it was both highly stable and repeatedly reached from other configurations. Interpreting this pattern simply as decline would nevertheless be too restrictive. The concept of *déprise* describes ageing as a selective reorganisation of activities and investments in relation to changing capacities, constraints and priorities rather than as uniform loss of engagement (Barthe et al., 1988; Caradec, 2022; Lalive d’Epinay et al., 2005). Some transitions towards Home-centred engagement may be compatible with such reorientation, but the present data cannot determine whether a particular trajectory reflects preference, adaptation, constraint or combinations of these processes.

This uncertainty also reflects the measurement boundary of the profiles. Home-centred engagement was characterised by relatively greater representation of individual and home-based measured activities and lower levels of several family, community and organised participation indicators. It does not measure the total amount, meaning or subjective value of everyday occupations. The analysis therefore cannot establish whether the home constitutes a chosen setting for engagement or the progressively narrowed boundary of what remains practicable. The same observed configuration may consequently represent very different biographical situations.

### 4.2 Life-course transitions and activity configurations

Not all longitudinal differences followed the pattern observed for functional deterioration. Retirement was associated with a lower probability of Home-centred engagement and higher probabilities of Community and, more modestly, Diversified engagement. This is consistent with recent SHARE-based evidence associating retirement with changes in volunteering, although these associations vary according to health, partnership and socioeconomic resources (Hämäläinen et al., 2024). More broadly, retirement is a life-course transition embedded in prior occupational, relational and socioeconomic trajectories rather than a uniform event with identical consequences for participation (Elder, 1994; Moen, 2016). The present findings therefore indicate that transition out of employment coincided with a different distribution of activity configurations, rather than establishing that retirement itself generated new participation opportunities.

Changes in household size similarly locate engagement within changing relational contexts. Both increases and decreases in household size were associated with a higher probability of Home-centred engagement, while an increase was additionally associated with Family-oriented engagement. This pattern is consistent with the life-course principle of linked lives, according to which individual trajectories unfold within relationships and shared living arrangements (Carr & Utz, 2020; Elder, 1994). Importantly, however, household-size change does not identify the biographical event involved. Bereavement, separation, the departure of a household member or the arrival of another person may entail very different relational, economic and practical circumstances. The indicator should therefore be interpreted as a change in the immediate co-residential context rather than as a proxy for any single family transition or caregiving process.

Taken together, these findings place changes in occupational engagement within broader life-course circumstances rather than functional health alone. Activity configurations varied alongside changes in employment status and co-residential circumstances as well as functional limitations. Because these dimensions were primarily examined in separate models and over the same wave intervals as profile change, their independent contributions and temporal ordering cannot be established; nevertheless, the contrasting patterns across these circumstances reinforce the interpretation of later-life engagement as differentiated change in multidomain configurations rather than a single directional process.

### 4.3 From individual activities to configurations of occupational engagement

A profile-based approach changes the unit of analysis from individual activities to their co-occurrence within everyday life. Its contribution is therefore not to identify fixed types of older adults, but to examine how participation domains combine and whether these configurations occupy different longitudinal positions. The marked differences in stability and transition patterns across profiles suggest that changes in later-life participation cannot be adequately represented by the maintenance or loss of isolated activities alone. This is consistent with occupational science approaches emphasising patterns of everyday activity rather than isolated behaviours (Eklund et al., 2017).

The profiles nevertheless remain analytical constructions. The five-profile solution represented a compromise across clustering diagnostics, and assignment to fixed W5 medoids introduced classification uncertainty. Longitudinal diagnostics suggested only modest increases in distance and ambiguity over time, supporting their use as common reference states without demonstrating structural invariance. More fundamentally, the profiles capture observable participation patterns but not meaning, identity, belonging or biographical significance. Profile analysis and activity-specific analysis should therefore be understood as complementary: the former captures how activities are organised together, while the latter retains detail on specific participation domains.

### 4.4 Strengths, limitations and implications

The study combines repeated observations from a large longitudinal sample with a multidomain representation of everyday activities and complementary analyses of stability, mobility and transition orientation. Fixed W5 medoids provided consistent longitudinal reference states, and sensitivity analyses included inverse probability weighting and diagnostics of longitudinal profile assignment.

Several limitations qualify the findings. First, the SHARE indicators capture only part of occupational engagement and incompletely represent domestic, informal and relational activities. The profiles therefore describe measured activity configurations rather than participants’ complete everyday engagement. Second, requiring wave 5 participation and consecutive observations, together with reduced CAPI follow-up during W8–W9, may have selected participants with more stable survey participation, against the broader pattern of differential participation and attrition documented across SHARE waves (Bergmann et al., 2025). IPW produced broadly similar results but cannot eliminate selection related to unmeasured characteristics. Missing data were not imputed, and the comparable economic deprivation measure was available for only a subset of wave intervals. Third, the five-profile solution and longitudinal medoid assignments involve classification uncertainty and should not be interpreted as natural categories. Finally, health, life-course and profile changes were measured over the same wave intervals, preventing temporal ordering. Most time-varying changes were also examined in separate models, so their associations cannot be interpreted as mutually adjusted independent effects, and residual confounding, reverse causation and reciprocal relationships remain possible.

For population health, these findings support complementing activity-specific indicators with attention to broader configurations of everyday engagement and to the functional and life-course circumstances accompanying their change. Importantly, Home-centred engagement should not be considered a priori as a less favourable form of ageing: similar observable configurations may have different significance depending on whether they reflect continuity, preference, adaptation or constrained opportunities for participation. This distinction shifts the implication from identifying a desirable profile towards understanding the conditions under which different forms of engagement remain possible. Future research combining engagement configurations with measures of environmental accessibility, resources, support, preferences and subjective meaning could help distinguish these processes and clarify where modifiable barriers to participation may arise.

## 5 Conclusion

Later-life engagement did not follow a uniform pattern of change. Among French participants in SHARE, five recurring configurations of reported everyday activities were identified, with marked differences in longitudinal stability and asymmetric transitions between profiles. Increases in functional limitations were associated with a higher probability of Home-centred engagement, whereas retirement and changes in household size were associated with different distributions of engagement configurations. Because these changes were observed over the same wave intervals, the findings describe concurrent associations rather than their temporal ordering. Taken together, the contrasting patterns across functional and life-course circumstances are consistent with differentiated change in multidomain engagement rather than a general progression towards more Home-centred engagement.

Examining activities as configurations therefore complements analyses focused on individual participation domains by showing how domains of everyday engagement combine and change over time. Home-centred engagement should not be interpreted as evidence of reduced engagement: similar observable configurations may reflect continuity, preference, adaptation or constrained opportunities under different circumstances. Understanding later-life engagement consequently requires attention not only to the activities people report, but also to the functional, relational and contextual conditions under which different configurations remain possible.

## CRediT authorship contribution statement

**Muriel Sahraoui:** Writing – review & editing, Writing – original draft, Visualization, Validation, Software, Methodology, Formal analysis, Data curation, Conceptualization. **Emmanuelle Cambois:** Writing – review & editing, Validation, Methodology. **Florence Canouï-Poitrine:** Writing – review & editing, Validation, Resources, Methodology. **Marie Laurent:** Supervision. **Meoïn Hagege:** Writing – review & editing, Supervision, Methodology.

## Code availability

Public deposition of the analysis code is planned, subject to agreement from all co-authors.

## Ethical statement

This study used secondary data from the Survey of Health, Ageing and Retirement in Europe (SHARE). SHARE is subject to continuous ethics review. Waves 1 to 4 were reviewed and approved by the Ethics Committee of the University of Mannheim, and Wave 4 and the continuation of the project were reviewed and approved by the Ethics Council of the Max Planck Society. Country implementations were also reviewed by the relevant ethics committees or institutional review boards whenever required. All SHARE participants provided informed consent before participation. The present study used anonymised research data made available to registered users under the SHARE conditions of use and did not require additional ethical approval.

## Declaration of generative AI and AI-assisted technologies in the manuscript preparation process

During the preparation of this work the authors used Antidote © in order to check spelling, grammar, punctuation, and syntax, without generating scientific content. After using this service, the authors reviewed and edited the content as needed and take full responsibility for the content of the published article.

## Funding

This research received no specific grant from any funding agency in the public, commercial, or not-for-profit sectors.

## Declaration of competing interest

The authors declare that they have no known competing financial interests or personal relationships that could have appeared to influence the work reported in this paper.

## Data Availability

Data are available from the Survey of Health, Ageing and Retirement in Europe (SHARE) upon registration and approval of access conditions.
