# Supplementary material for: Longitudinal change in occupational engagement configurations in later life: evidence from SHARE France
Source: SSM Popul Health. 2026 Oct 24;36:101990. doi: 10.1016/j.ssmph.2026.101990 (PMC13722054; doi:10.1016/j.ssmph.2026.101990)
Supplement: Supplementary file 1 [file mmc1.docx]

**Supplementary Table S1. Clustering diagnostics for solutions ranging from two to ten profiles at W5**

| Number of profiles | ASW | CH | DB | R² | PBM |
| --- | --- | --- | --- | --- | --- |
| 2 | 0.512 | 342.009 | 3.055 | 0.18 | 9.858 |
| 3 | 0.323 | 458.401 | 2.740 | 0.27 | 9.488 |
| 4 | 0.367 | 459.231 | 2.586 | 0.35 | 9.039 |
| **5** | **0.412** | **482.750** | **2.644** | **0.43** | **6.359** |
| 6 | 0.318 | 361.426 | 2.955 | 0.48 | 5.352 |
| 7 | 0.199 | 283.051 | 3.228 | 0.50 | 4.054 |
| 8 | 0.215 | 244.684 | 3.216 | 0.53 | 4.420 |
| 9 | 0.205 | 256.122 | 3.170 | 0.54 | 3.675 |
| 10 | 0.188 | 226.058 | 3.140 | 0.54 | 3.079 |

**Note.** ASW = Average Silhouette Width; CH = Calinski–Harabasz index; DB = Davies–Bouldin index; PBM = Pakhira–Bandyopadhyay–Maulik index. Higher values of ASW, CH, R² and PBM generally indicate better solutions, whereas lower DB values indicate better separation. No single criterion identified an unequivocally optimal solution. The five-profile solution was retained as a compromise between statistical quality, empirical differentiation and interpretability for longitudinal follow-up. These are the diagnostic values used for the final five-profile solution.

**Supplementary Table S2. Distance to W5 reference medoids and assignment ambiguity across waves**

| **Wave** | **N** | **d₁, median [IQR]** | **d₁/d₂, median [IQR]** | **P90 d₁/d₂** |
| --- | --- | --- | --- | --- |
| W5 | 4,505 | 0.142 [0.101–0.191] | 0.61 [0.43–0.78] | 0.91 |
| W6 | 4,505 | 0.148 [0.105–0.198] | 0.63 [0.45–0.80] | 0.92 |
| W7 | 4,505 | 0.153 [0.109–0.205] | 0.64 [0.46–0.81] | 0.93 |
| W8 | 2,603 | 0.157 [0.112–0.211] | 0.65 [0.47–0.82] | 0.93 |
| W9 | 1,937 | 0.160 [0.114–0.216] | 0.66 [0.48–0.83] | 0.94 |

**Note.** d₁ is the Gower distance to the closest W5 reference medoid and d₂ the distance to the second-closest medoid. The d₁/d₂ ratio indicates the relative separation between the two closest assignments; values closer to 1 indicate a less clearly differentiated assignment. IQR = interquartile range; P90 = 90th percentile.

**Supplementary Table S3. Distribution of occupational engagement profiles across waves contributing to consecutive transitions**

| Wave | P1 Home-centred engagement | P2 Family-oriented engagement | P3 Community engagement | P4 Diversified engagement | P5 Social engagement | Total |
| --- | --- | --- | --- | --- | --- | --- |
| W5 | 3,298 (73.2%) | 440 (9.8%) | 171 (3.8%) | 344 (7.6%) | 252 (5.6%) | 4,505 |
| W6 | 3,184 (70.7%) | 385 (8.5%) | 384 (8.5%) | 333 (7.4%) | 219 (4.9%) | 4,505 |
| W7 | 3,719 (82.6%) | 298 (6.6%) | 185 (4.1%) | 159 (3.5%) | 144 (3.2%) | 4,505 |
| W8 | 1,926 (74.0%) | 131 (5.0%) | 61 (2.3%) | 306 (11.8%) | 179 (6.9%) | 2,603 |
| W9 | 1,336 (73.6%) | 98 (5.4%) | 50 (2.8%) | 233 (12.8%) | 97 (5.4%) | 1,814 |

**Note.** Values are n (row %). Counts correspond to observations contributing to consecutive-wave transitions. W5 represents profiles at the origin of the W5→W6 transition; W6–W9 represent profiles observed at the destination of successive transitions. At W9, 1,937 profiles were available overall, of which 1,814 also had a profile at W8 and could therefore contribute to the W8→W9 transition.

**Supplementary Table S4. Characteristics of complete occupational engagement sequences from W5–W9**

| Profile at W5 | N | Distinct states, median [IQR] | Changes, median [IQR] | Fully stable sequence, n (%) | Entropy, median [IQR] |
| --- | --- | --- | --- | --- | --- |
| P1 Home-centred engagement | 1,205 | 2 [1–2] | 1 [0–2] | 612 (50.8%) | 0.311 [0–0.418] |
| P2 Family-oriented engagement | 230 | 3 [2–3] | 2 [1–3] | 2 (0.9%) | 0.590 [0.418–0.655] |
| P3 Community engagement | 86 | 3 [3–4] | 2 [2–3] | 0 (0%) | 0.655 [0.590–0.828] |
| P4 Diversified engagement | 192 | 3 [3–3] | 2 [2–3] | 0 (0%) | 0.590 [0.590–0.655] |
| P5 Social engagement | 101 | 3 [3–4] | 2 [2–3] | 0 (0%) | 0.590 [0.590–0.828] |
| Overall | 1,814 | 2 [1–3] | 1 [0–2] | 614 (33.8%) | 0.418 [0–0.590] |

**Note.** Analysis restricted to participants with an occupational engagement profile at all five waves. Distinct states indicate the number of different profiles occupied over the sequence. Changes indicate movements from one profile to another between consecutive waves. A fully stable sequence indicates remaining in the same profile at all five waves. Normalised entropy ranges from 0, for a sequence entirely concentrated in one state, to 1, for maximum diversity of states over follow-up.

**Supplementary Table S5. Average marginal effects associated with entry into Family-oriented engagement**

| Variable | AME (pp) | 95% CI | p |
| --- | --- | --- | --- |
| Sex |  |  |  |
| Women vs men | +1.70 | [0.13, 3.27] | .034 |
| Age, +1 year | −0.12 | [−0.21, −0.04] | .004 |
| ADL limitations, +1 | −1.75 | [−3.34, −0.16] | .031 |
| IADL limitations, +1 | −3.51 | [−4.49, −2.53] | <.001 |
| Profile at origin |  |  |  |
| P1 Home-centred engagement | Ref. | — | — |
| P3 Community engagement | −5.73 | [−9.03, −2.42] | .001 |
| P4 Diversified engagement | −0.89 | [−3.82, 2.05] | .554 |
| P5 Social engagement | −6.07 | [−8.89, −3.26] | <.001 |
| Wave at origin |  |  |  |
| W5 | Ref. | — | — |
| W6 | −39.36 | [−42.13, −36.60] | <.001 |
| W7 | −49.51 | [−51.62, −47.40] | <.001 |
| W8 | −49.87 | [−52.04, −47.71] | <.001 |

**Note.** AMEs are expressed in percentage points. The dependent variable was entry into P2 Family-oriented engagement at the following wave among transitions originating from any profile other than P2. The logistic model was adjusted for sex, age, number of ADL and IADL limitations, origin profile and origin wave. Robust standard errors were clustered at the individual level. N = 11,331 transitions among 4,271 participants.

**Supplementary Table S6. Inverse probability weighting sensitivity analyses**

**Panel A. Construction and distribution of stabilised inverse probability weights**

| Indicator | W5→W6 | W6→W7 | W7→W8 | W8→W9 |
| --- | --- | --- | --- | --- |
| Observations with profile at t | 4,505 | 4,505 | 4,505 | 2,603 |
| Observed at t+1, n (%) | 4,505 (100.0%) | 4,505 (100.0%) | 2,603 (57.8%) | 1,814 (69.7%) |
| N in response model | — | — | 4,372 | 2,525 |
| Transitions with available weight | 4,505 | 4,505 | 2,525 | 1,765 |
| Weight type | 1 | 1 | Stabilised | Cumulative stabilised |
| Mean | 1.000 | 1.000 | 0.994 | 0.954 |
| SD | 0.000 | 0.000 | 0.409 | 0.671 |
| Median | 1.000 | 1.000 | 0.734 | 0.691 |
| 99th percentile | 1.000 | 1.000 | 2.098 | 3.251 |
| Maximum | 1.000 | 1.000 | 3.530 | 15.523 |
| Weight >10, n | 0 | 0 | 0 | 1 |

**Note.** The probability of remaining observed at the following wave was estimated for W7→W8 and W8→W9 using age, sex, education, baseline ADL and IADL limitations, occupational engagement profile at the origin wave and self-rated health at that wave. Weights were stabilised using the marginal probability of remaining under follow-up. For W8→W9, the weight was the product of the stabilised weights for W7→W8 and W8→W9. W5→W6 and W6→W7 were assigned a weight of 1. No weight truncation was applied.

**Panel B. Sensitivity analysis of overall profile mobility**

| Variable | Unweighted β (SE) | IPW β (SE) |
| --- | --- | --- |
| Origin profile |  |  |
| P1 Home-centred engagement | Ref. | Ref. |
| P2 Family-oriented engagement | 2.344 (0.094) | 2.354 (0.098) |
| P3 Community engagement | 2.600 (0.146) | 2.519 (0.153) |
| P4 Diversified engagement | 2.037 (0.095) | 2.026 (0.100) |
| P5 Social engagement | 3.078 (0.149) | 3.042 (0.158) |
| Age, +1 year | −0.006 (0.003) | −0.007 (0.003) |
| Women vs men | −0.140 (0.051) | −0.135 (0.053) |
| Education |  |  |
| Level 1 | Ref. | Ref. |
| Level 2 | 0.137 (0.061) | 0.135 (0.062) |
| Level 3 | 0.225 (0.072) | 0.247 (0.075) |
| Level 4 | 0.167 (0.142) | 0.153 (0.149) |
| ADL limitations, +1 | −0.123 (0.044) | −0.127 (0.043) |
| IADL limitations, +1 | −0.340 (0.032) | −0.349 (0.031) |
| Origin wave |  |  |
| W5 | Ref. | Ref. |
| W6 | −0.370 (0.078) | −0.363 (0.079) |
| W7 | −1.304 (0.068) | −1.364 (0.069) |
| W8 | −1.562 (0.077) | −1.676 (0.079) |
| Constant | 0.584 (0.204) | 0.627 (0.208) |

Note. N = 11,211 transitions with complete covariate and IPW information. Unweighted and IPW models were estimated on the same analytical sample, with standard errors clustered at the individual level. Values are logistic regression coefficients β with standard errors in parentheses. The primary analysis of overall mobility remains the marginal GEE model reported in the main text.

**Panel C. Comparison of unweighted and IPW average marginal effects for transition orientation**

| Change | Destination profile | Unweighted AME, pp (p) | IPW AME, pp (p) |
| --- | --- | --- | --- |
| Deterioration in self-rated health N = 9,072 | P1 Home-centred | +7.61 (<.001) | +7.01 (<.001) |
|  | P2 Family-oriented | −0.36 (.764) | +0.06 (.958) |
|  | P3 Community | −2.14 (.002) | −2.25 (.001) |
|  | P4 Diversified | −3.41 (<.001) | −3.27 (<.001) |
|  | P5 Social | −1.70 (.010) | −1.55 (.017) |
| Increase in ADL limitations N = 9,044 | P1 Home-centred | +17.76 (<.001) | +19.30 (<.001) |
|  | P2 Family-oriented | −7.22 (<.001) | −9.00 (<.001) |
|  | P3 Community | −3.15 (<.001) | −3.42 (<.001) |
|  | P4 Diversified | −5.31 (<.001) | −4.86 (<.001) |
|  | P5 Social | −2.07 (.009) | −2.01 (.008) |
| Increase in IADL limitations N = 9,050 | P1 Home-centred | +22.37 (<.001) | +24.39 (<.001) |
|  | P2 Family-oriented | −10.37 (<.001) | −12.28 (<.001) |
|  | P3 Community | −2.69 (<.001) | −3.11 (<.001) |
|  | P4 Diversified | −6.02 (<.001) | −5.73 (<.001) |
|  | P5 Social | −3.28 (<.001) | −3.27 (<.001) |
| Transition from employment to retirement N = 8,844 | P1 Home-centred | −12.76 (<.001) | −13.22 (<.001) |
|  | P2 Family-oriented | −0.18 (.931) | +0.93 (.670) |
|  | P3 Community | +8.17 (<.001) | +8.76 (<.001) |
|  | P4 Diversified | +3.97 (.017) | +2.92 (.058) |
|  | P5 Social | +0.80 (.525) | +0.61 (.616) |
| Decrease in household size N = 13,300 | P1 Home-centred | +7.21 (<.001) | +7.81 (<.001) |
|  | P2 Family-oriented | −3.00 (.033) | −3.78 (.007) |
|  | P3 Community | −2.22 (.002) | −2.23 (.002) |
|  | P4 Diversified | −2.00 (.034) | −1.56 (.086) |
|  | P5 Social | +0.01 (.987) | −0.23 (.770) |
| Increase in household size N = 13,300 | P1 Home-centred | +38.45 (<.001) | +37.94 (<.001) |
|  | P2 Family-oriented | +9.80 (.019) | +9.61 (<.001) |
|  | P3 Community | −15.19 (<.001) | −14.88 (<.001) |
|  | P4 Diversified | −18.12 (<.001) | −18.16 (<.001) |
|  | P5 Social | −14.93 (<.001) | −14.52 (<.001) |
| Increase in economic difficulties N = 3,464 | P1 Home-centred | −2.33 (.345) | −1.78 (.499) |
|  | P2 Family-oriented | −1.78 (.673) | −2.57 (.561) |
|  | P3 Community | +0.82 (.754) | +2.84 (.375) |
|  | P4 Diversified | +3.44 (.125) | +2.05 (.343) |
|  | P5 Social | −0.15 (.930) | −0.54 (.747) |

**Note.** AMEs are expressed in percentage points. For each change indicator, weighted and unweighted models were estimated on the same analytical sample and adjusted for the origin profile. The model for economic difficulties was additionally adjusted for origin wave and was restricted to W5→W6 and W8→W9, the only intervals for which the economic score could be constructed from the same potential set of eight items. Standard errors were clustered at the individual level. The IPW estimates are sensitivity analyses rather than the primary estimates.
